# Crystal structure of *S*-octyl (*E*)-3-(4-meth­oxy­benzyl­idene)di­thio­carbazate

**DOI:** 10.1107/S205698901500568X

**Published:** 2015-03-28

**Authors:** M. S. Begum, E. Zangrando, M. C. Sheikh, R. Miyatake, M. M. Hossain

**Affiliations:** a Department of Chemistry, Rajshahi University, Rajshahi-6205, Bangladesh; b Department of Chemical and Pharmaceutical Sciences, via Giorgieri 1, 34127 Trieste, Italy; c Department of Applied Chemistry, Faculty of Engineering, University of Toyama, 3190 Gofuku, Toyama 930-8555, Japan; d Center for Environmental Conservation and Research Safety, University of Toyama, 3190 Gofuku, Toyama 930-8555, Japan

**Keywords:** crystal structure, di­thio­carbazate, S-containing Schiff bases, hydrogen bonding

## Abstract

As already observed in similar mol­ecules, the di­thio­carbazate group in the title compound, C_17_H_26_N_2_OS_2_, adopts an *EE* configuration with respect to the C=N bond of the benzyl­idene moiety. In the crystal, mol­ecules are connected into inversion dimers by pairs of N—H⋯S hydrogen bonds. The dimers are linked by weak π–π inter­actions, with centroid-to-centroid distances of 3.723 (11) Å, forming chains parallel to [110].

## Related literature   

For the structures of related compounds, see: Howlader *et al.* (2015 ▸); Begum *et al.* (2015 ▸). For metal complexes containing similar ligands, see: Chan *et al.* (2008 ▸); How *et al.* (2008 ▸); Tarafder *et al.* (2002 ▸); Ali *et al.* (2002 ▸); Chew *et al.* (2004 ▸); Crouse *et al.* (2004 ▸).
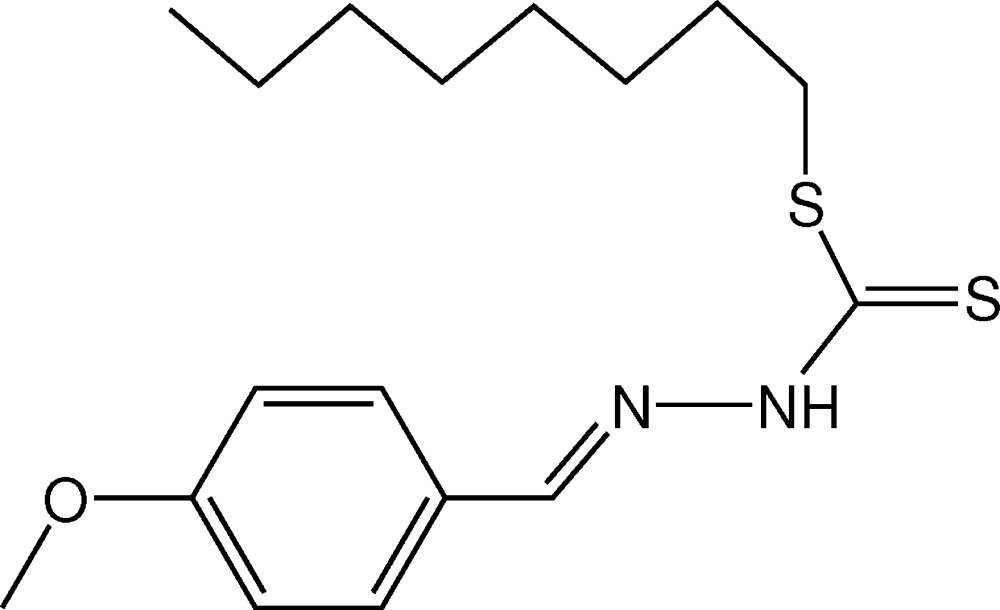


## Experimental   

### Crystal data  

C_17_H_26_N_2_OS_2_
*M* *_r_* = 338.53
Monoclinic, 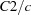

*a* = 28.7970 (6) Å
*b* = 8.37150 (15) Å
*c* = 15.6207 (3) Å
β = 104.2210 (7)°
*V* = 3650.36 (12) Å^3^
*Z* = 8
Cu *K*α radiation
μ = 2.66 mm^−1^
*T* = 173 K
0.32 × 0.21 × 0.13 mm

### Data collection  

Rigaku R-AXIS RAPID diffractometer
Absorption correction: multi-scan (*ABSCOR*; Rigaku, 1995 ▸) *T*_min_ = 0.581, *T*_max_ = 0.708
20438 measured reflections
3333 independent reflections
3140 reflections with *F* ^2^ > 2σ(*F* ^2^)
*R*_int_ = 0.056

### Refinement  

*R*[*F* ^2^ > 2σ(*F* ^2^)] = 0.035
*wR*(*F* ^2^) = 0.096
*S* = 1.06
3333 reflections
205 parameters
H atoms treated by a mixture of independent and constrained refinement
Δρ_max_ = 0.37 e Å^−3^
Δρ_min_ = −0.33 e Å^−3^

### 

Data collection: *RAPID-AUTO* (Rigaku, 2001 ▸); cell refinement: *RAPID-AUTO*; data reduction: *RAPID-AUTO*; program(s) used to solve structure: *SIR92* (Altomare *et al.*, 1994 ▸); program(s) used to refine structure: *SHELXL97* (Sheldrick, 2008 ▸); molecular graphics: *CrystalStructure* (Rigaku, 2010 ▸); software used to prepare material for publication: *CrystalStructure*.

## Supplementary Material

Crystal structure: contains datablock(s) General, I. DOI: 10.1107/S205698901500568X/rz5151sup1.cif

Structure factors: contains datablock(s) I. DOI: 10.1107/S205698901500568X/rz5151Isup2.hkl

Supporting information file. DOI: 10.1107/S205698901500568X/rz5151Isup3.cml

**Fig. 1.**
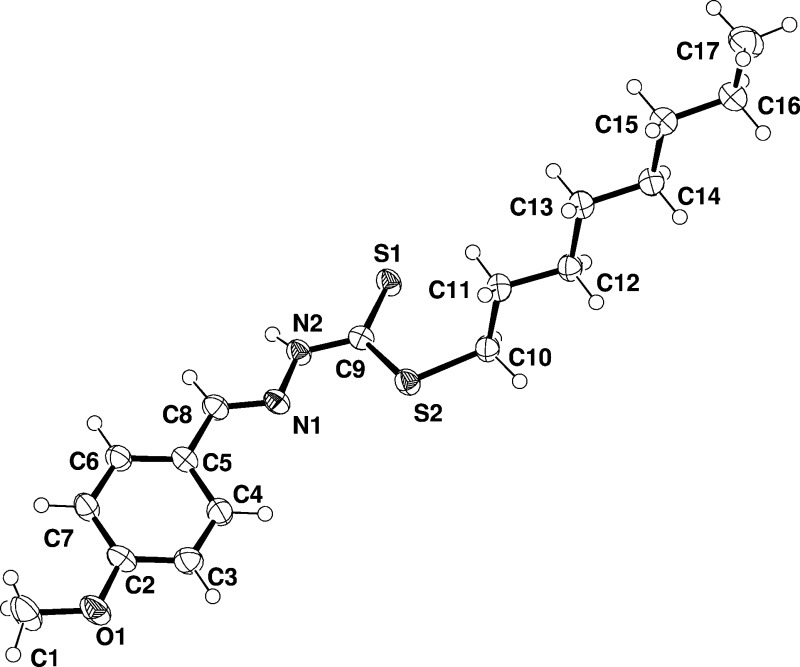
The molecular structure of the title compound with displacement ellipsoids drawn at the 50% probability level.

. DOI: 10.1107/S205698901500568X/rz5151fig1.tif
The mol­ecular structure of the title compound with displacement ellipsoids drawn at the 50% probability level.

**Fig. 2.**
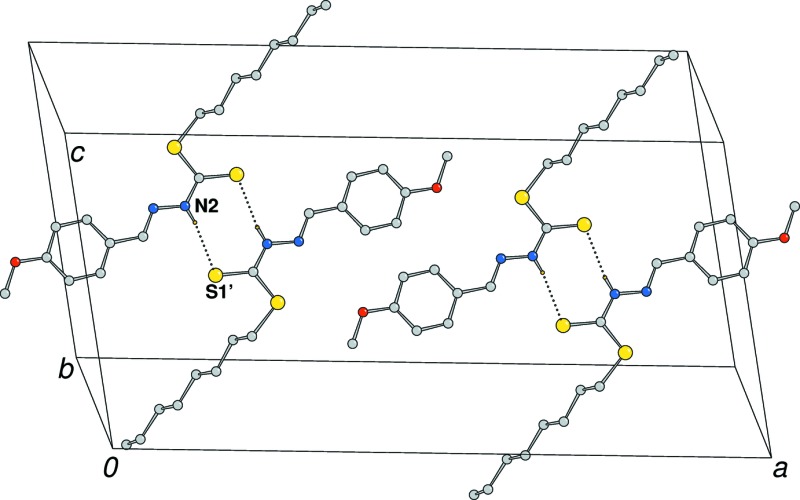
Crystal packing of the title compound showing pairs of molecules connected by N—H···S hydrogen interactions (dashed lines). H atoms not involved in hydrogen bonding are omitted.

. DOI: 10.1107/S205698901500568X/rz5151fig2.tif
Crystal packing of the title compound showing pairs of mol­ecules connected by N—H⋯S hydrogen inter­actions (dashed lines). H atoms not involved in hydrogen bonding are omitted.

CCDC reference: 1044476

Additional supporting information:  crystallographic information; 3D view; checkCIF report

## Figures and Tables

**Table 1:** Hydrogen-bond geometry (, )

*D*H*A*	*D*H	H*A*	*D* *A*	*D*H*A*
N2H9S1^i^	0.86(2)	2.536(18)	3.3870(11)	170.9(16)

Symmetry code: (i) 
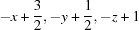
.

## supplementary crystallographic information

### S1. Chemical context

Bidentate Schiff bases of S-methyl or S-benzyl di­thio­carbaza­tes and their metal complexes have received considerable attention for their possible bioactivities (Chan *et al.*, 2008; How *et al.*, 2008; Tarafder *et al.*, 2002; Ali *et al.*, 2002; Chew *et al.*, 2004; Crouse *et al.*, 2004). As part of our ongoing structural studies of S-containing Schiff bases (Howlader *et al.*, 2015; Begum *et al.*, 2015), we report herein the structure of the title compound having a long alkyl chain.

### S2. Structural commentary

The molecule of the title compound is shown in Fig. 1. The Schiff base exists in thione tautomeric form with the di­thio­carbazate fragment adopting an *EE* configuration with respect to the C═N bond of the benzyl­idene moiety. The β-nitro­gen and the thio­keto sulphur are *trans* located with respect to the C9–N2 bond. With the exception of the S-octyl chain, the atoms of the 3-(4-meth­oxy­benzyl­idene)di­thio­carbazate group are approximately co-planar (r.m.s. deviation = 0.253 Å) indicating electron delocalization within it. The bond lengths and angles are closely comparable to those detected in S-hexyl (*E*)-3-(4-methyl­benzyl­idene)di­thio­carbazate (Howlader *et al.*, 2015) and in the S-hexyl analogue (Begum *et al.*, 2015), characterized by a shorter alkyl chain.

### S3. Supra­molecular features

The crystal packing of the title compound evidences molecules connected into centrosymmetric dimers (Fig. 2) by pairs of N—H···S hydrogen bonds (Table 1), which are further linked by weak π-π stacking inter­actions (centroid-to-centroid distances of 3.723 (11) Å) to form chains parallel to the [1 1 0] direction.

### S4. Database survey

The octyl chain shows the typical all-*anti* conformation with a S(2)—C(10)—C(11)—C(12) torsion angle of 169.73 (9)°, which differs from that of 66.6 (2)° measured in the corresponding S-hexyl (*E*)-3-(4-methyl­benzyl­idene)di­thio­carbazate (Howlader *et al.*, 2015), but comparable to the value of 173.99 (13)° found in the hexyl derivative (Begum *et al.*, 2015) .

### S5. Synthesis and crystallization

To an ethano­lic solution of KOH (2.81 g, 0.05 mol) hydrazine hydrate (2.50 g, 0.05 mol, 99%) was added and the mixture was stirred at 273 K. To this solution carbon di­sulfide (3.81 g, 0.05 mol) was added dropwise with constant stirring for one hour. Then 1-bromo­octane (9.65 g, 0.05 mol) was added dropwise with vigorous stirring at 273 K for an additional hour. Finally, 4-meth­oxy­benzaldehyde (6.81 g, 0.05 mol) in ethanol was added and the mixture refluxed for 30 min. The mixture was filtered while hot and then the filtrate was cooled to 273 K giving a precipitate of the Schiff base product, which was recrystallized from ethanol at room temperature and dried in a vacuum desiccator over anhydrous CaCl_2_. Colourless crystals, suitable for X-ray diffraction of the compound were obtained by slow evaporation of an ethanol/aceto­nitrile (2:1 *v*/*v*) solution after 19 days (m. p. 355 K).

### S6. Refinement

Hydrogen atoms were located geometrically and treated as riding atoms with C—H = 0.98–0.99 Å and *U*_ĩso_(H) = 1.2*U*_eq_(C). The hydrogen atom at N2 was located on the difference Fourier map and freely refined.

### Crystal data

**Table:**

C_17_H_26_N_2_OS_2_	*F*(000) = 1456.00
*M**_r_* = 338.53	*D*_x_ = 1.232 Mg m^−^^3^
Monoclinic, *C*2/*c*	Cu *K*α radiation, λ = 1.54187 Å
Hall symbol: -C 2yc	Cell parameters from 19039 reflections
*a* = 28.7970 (6) Å	θ = 3.2–68.2°
*b* = 8.37150 (15) Å	µ = 2.66 mm^−^^1^
*c* = 15.6207 (3) Å	*T* = 173 K
β = 104.2210 (7)°	Prism, colorless
*V* = 3650.36 (12) Å^3^	0.32 × 0.21 × 0.13 mm
*Z* = 8	

### Data collection

**Table:**

Rigaku R-AXIS RAPID diffractometer	3140 reflections with *F*^2^ > 2σ(*F*^2^)
Detector resolution: 10.000 pixels mm^-1^	*R*_int_ = 0.056
ω scans	θ_max_ = 68.2°
Absorption correction: multi-scan (*ABSCOR*; Rigaku, 1995)	*h* = −34→34
*T*_min_ = 0.581, *T*_max_ = 0.708	*k* = −10→9
20438 measured reflections	*l* = −18→18
3333 independent reflections	

### Refinement

**Table:**

Refinement on *F*^2^	Secondary atom site location: difference Fourier map
*R*[*F*^2^ > 2σ(*F*^2^)] = 0.035	Hydrogen site location: inferred from neighbouring sites
*wR*(*F*^2^) = 0.096	H atoms treated by a mixture of independent and constrained refinement
*S* = 1.06	*w* = 1/[σ^2^(*F*_o_^2^) + (0.0604*P*)^2^ + 1.4685*P*] where *P* = (*F*_o_^2^ + 2*F*_c_^2^)/3
3333 reflections	(Δ/σ)_max_ = 0.002
205 parameters	Δρ_max_ = 0.37 e Å^−^^3^
0 restraints	Δρ_min_ = −0.33 e Å^−^^3^
Primary atom site location: structure-invariant direct methods	

### Special details

**Table:**

Geometry. ENTER SPECIAL DETAILS OF THE MOLECULAR GEOMETRY
Refinement. Refinement was performed using all reflections. The weighted *R*-factor (*wR*) and goodness of fit (*S*) are based on *F*^2^. *R*-factor (gt) are based on *F*. The threshold expression of *F*^2^ > 2.0 σ(*F*^2^) is used only for calculating *R*-factor (gt).

### Fractional atomic coordinates and isotropic or equivalent isotropic displacement parameters (Å^2^)

**Table:**

	*x*	*y*	*z*	*U*_iso_*/*U*_eq_	
S2	0.807022 (11)	0.14416 (4)	0.28621 (2)	0.03085 (13)	
S1	0.719578 (11)	0.16904 (4)	0.36276 (2)	0.03091 (13)	
O1	1.08208 (4)	0.33645 (14)	0.60247 (7)	0.0427 (3)	
N1	0.85671 (4)	0.24695 (14)	0.45179 (7)	0.0295 (3)	
N2	0.80783 (4)	0.24227 (14)	0.44385 (7)	0.0290 (3)	
C1	1.10812 (5)	0.4216 (3)	0.67848 (11)	0.0535 (5)	
C2	1.03338 (5)	0.33880 (18)	0.58554 (9)	0.0331 (4)	
C3	1.00927 (5)	0.24210 (19)	0.51590 (9)	0.0376 (4)	
C4	0.96000 (5)	0.23336 (18)	0.49421 (9)	0.0349 (4)	
C5	0.93337 (5)	0.32172 (16)	0.54197 (9)	0.0288 (3)	
C6	0.95789 (5)	0.41699 (18)	0.61083 (9)	0.0327 (3)	
C7	1.00768 (5)	0.42693 (18)	0.63288 (9)	0.0344 (4)	
C8	0.88118 (5)	0.31449 (17)	0.52194 (9)	0.0298 (3)	
C9	0.77823 (5)	0.18870 (16)	0.36983 (9)	0.0272 (3)	
C10	0.75805 (5)	0.08849 (17)	0.19384 (8)	0.0304 (3)	
C11	0.73334 (5)	0.23027 (17)	0.14066 (9)	0.0315 (3)	
C12	0.69859 (5)	0.17876 (17)	0.05459 (9)	0.0312 (3)	
C13	0.67214 (5)	0.32009 (17)	0.00317 (9)	0.0335 (4)	
C14	0.63553 (5)	0.27125 (18)	−0.08091 (9)	0.0342 (3)	
C15	0.61160 (5)	0.41321 (19)	−0.13503 (9)	0.0362 (4)	
C16	0.57411 (6)	0.3675 (2)	−0.21842 (10)	0.0405 (4)	
C17	0.55655 (6)	0.5106 (3)	−0.27733 (11)	0.0526 (5)	
H1	1.0974	0.3871	0.7304	0.0641*	
H2	1.1024	0.5364	0.6694	0.0641*	
H3	1.1424	0.3995	0.6878	0.0641*	
H4	1.0270	0.1819	0.4832	0.0451*	
H5	0.9439	0.1672	0.4467	0.0419*	
H6	0.9403	0.4770	0.6438	0.0393*	
H7	1.0238	0.4937	0.6800	0.0413*	
H8	0.8650	0.3610	0.5619	0.0358*	
H9	0.7974 (6)	0.265 (2)	0.4894 (12)	0.034 (5)*	
H10	0.7705	0.0170	0.1542	0.0365*	
H11	0.7341	0.0276	0.2164	0.0365*	
H12	0.7579	0.3020	0.1269	0.0378*	
H13	0.7156	0.2912	0.1767	0.0378*	
H14	0.7166	0.1221	0.0173	0.0374*	
H15	0.6749	0.1032	0.0682	0.0374*	
H16	0.6555	0.3797	0.0417	0.0402*	
H17	0.6958	0.3930	−0.0126	0.0402*	
H18	0.6517	0.2055	−0.1177	0.0410*	
H19	0.6106	0.2043	−0.0649	0.0410*	
H20	0.6366	0.4788	−0.1518	0.0434*	
H21	0.5962	0.4801	−0.0976	0.0434*	
H22	0.5880	0.2879	−0.2519	0.0486*	
H23	0.5465	0.3170	−0.2017	0.0486*	
H24	0.5455	0.5939	−0.2429	0.0631*	
H25	0.5300	0.4779	−0.3265	0.0631*	
H26	0.5828	0.5524	−0.3006	0.0631*	

### Atomic displacement parameters (Å^2^)

**Table:**

	*U*^11^	*U*^22^	*U*^33^	*U*^12^	*U*^13^	*U*^23^
S2	0.0245 (2)	0.0413 (3)	0.0272 (2)	−0.00063 (13)	0.00700 (14)	−0.00084 (12)
S1	0.02225 (19)	0.0414 (3)	0.0290 (2)	−0.00425 (12)	0.00629 (14)	−0.00176 (13)
O1	0.0221 (5)	0.0673 (8)	0.0384 (6)	0.0004 (5)	0.0065 (5)	0.0005 (5)
N1	0.0217 (6)	0.0356 (7)	0.0302 (6)	−0.0007 (5)	0.0047 (5)	0.0028 (5)
N2	0.0218 (6)	0.0381 (7)	0.0271 (6)	−0.0021 (5)	0.0058 (5)	−0.0005 (5)
C1	0.0243 (7)	0.0921 (15)	0.0420 (9)	−0.0058 (8)	0.0044 (7)	−0.0050 (9)
C2	0.0230 (7)	0.0447 (9)	0.0309 (8)	0.0007 (6)	0.0053 (6)	0.0091 (6)
C3	0.0313 (8)	0.0473 (9)	0.0353 (8)	0.0050 (7)	0.0105 (6)	−0.0007 (7)
C4	0.0306 (7)	0.0411 (9)	0.0318 (7)	−0.0000 (6)	0.0052 (6)	−0.0029 (6)
C5	0.0243 (7)	0.0332 (8)	0.0279 (7)	−0.0010 (6)	0.0044 (5)	0.0052 (6)
C6	0.0259 (7)	0.0420 (9)	0.0299 (7)	0.0018 (6)	0.0060 (6)	−0.0009 (6)
C7	0.0266 (7)	0.0433 (9)	0.0306 (7)	−0.0024 (6)	0.0018 (6)	−0.0012 (6)
C8	0.0254 (7)	0.0347 (8)	0.0295 (7)	−0.0004 (6)	0.0068 (6)	0.0031 (6)
C9	0.0260 (7)	0.0273 (7)	0.0282 (7)	−0.0002 (5)	0.0069 (5)	0.0037 (5)
C10	0.0310 (7)	0.0329 (8)	0.0271 (7)	−0.0015 (6)	0.0064 (6)	−0.0030 (6)
C11	0.0338 (7)	0.0318 (8)	0.0278 (7)	−0.0028 (6)	0.0056 (6)	−0.0008 (6)
C12	0.0313 (7)	0.0349 (8)	0.0272 (7)	−0.0016 (6)	0.0070 (6)	−0.0024 (6)
C13	0.0331 (7)	0.0360 (8)	0.0302 (7)	−0.0017 (6)	0.0055 (6)	−0.0016 (6)
C14	0.0335 (8)	0.0366 (8)	0.0311 (7)	−0.0005 (6)	0.0053 (6)	−0.0021 (6)
C15	0.0347 (8)	0.0376 (9)	0.0351 (8)	0.0016 (6)	0.0064 (6)	−0.0009 (6)
C16	0.0372 (9)	0.0445 (9)	0.0360 (8)	0.0041 (7)	0.0020 (7)	0.0003 (7)
C17	0.0503 (10)	0.0593 (12)	0.0443 (9)	0.0100 (9)	0.0043 (8)	0.0105 (8)

### Geometric parameters (Å, º)

**Table:**

S2—C9	1.7506 (16)	C1—H2	0.980
S2—C10	1.8133 (13)	C1—H3	0.980
S1—C9	1.6734 (15)	C3—H4	0.950
O1—C1	1.429 (2)	C4—H5	0.950
O1—C2	1.3619 (19)	C6—H6	0.950
N1—N2	1.3829 (17)	C7—H7	0.950
N1—C8	1.2797 (17)	C8—H8	0.950
N2—C9	1.3343 (16)	C10—H10	0.990
C2—C3	1.396 (2)	C10—H11	0.990
C2—C7	1.381 (3)	C11—H12	0.990
C3—C4	1.377 (2)	C11—H13	0.990
C4—C5	1.405 (3)	C12—H14	0.990
C5—C6	1.3851 (19)	C12—H15	0.990
C5—C8	1.459 (2)	C13—H16	0.990
C6—C7	1.392 (2)	C13—H17	0.990
C10—C11	1.5214 (19)	C14—H18	0.990
C11—C12	1.5277 (18)	C14—H19	0.990
C12—C13	1.525 (2)	C15—H20	0.990
C13—C14	1.5242 (18)	C15—H21	0.990
C14—C15	1.522 (2)	C16—H22	0.990
C15—C16	1.5224 (19)	C16—H23	0.990
C16—C17	1.520 (3)	C17—H24	0.980
N2—H9	0.860 (19)	C17—H25	0.980
C1—H1	0.980	C17—H26	0.980
			
S2···N1	2.7676 (11)	C17···H1^viii^	3.3558
S1···C10	3.1750 (15)	C17···H5^x^	3.0329
N1···C4	2.8863 (18)	C17···H23^ix^	3.3270
C1···C7	2.804 (2)	C17···H24^ix^	3.1111
C2···C5	2.796 (2)	C17···H25^ix^	3.3030
C3···C6	2.759 (3)	H1···C6^xii^	3.2687
C4···C7	2.7824 (19)	H1···C17^iii^	3.3558
C8···C9	3.4760 (18)	H1···H6^xii^	2.5789
C9···C11	3.5119 (19)	H1···H8^xii^	3.1650
S1···N2^i^	3.3870 (13)	H1···H22^xiii^	3.3830
O1···C13^ii^	3.581 (2)	H1···H24^iii^	2.9558
O1···C17^iii^	3.490 (3)	H1···H26^iii^	2.8571
N2···S1^i^	3.3870 (13)	H2···N1^iv^	3.0576
C2···C17^iii^	3.447 (3)	H2···C4^iv^	3.3491
C3···C7^iv^	3.572 (2)	H2···C5^iv^	3.4202
C7···C3^iv^	3.572 (2)	H2···C8^iv^	3.3768
C9···C10^v^	3.574 (2)	H2···C16^xiii^	3.4817
C10···C9^vi^	3.574 (2)	H2···H5^iv^	3.1680
C13···O1^vii^	3.581 (2)	H2···H6^xii^	3.4703
C17···O1^viii^	3.490 (3)	H2···H15^ii^	3.1378
C17···C2^viii^	3.447 (3)	H2···H18^xiii^	3.5729
C17···C17^ix^	3.564 (3)	H2···H22^xiii^	2.5239
S2···H9	3.408 (19)	H3···S1^ii^	3.1229
S2···H12	2.8652	H3···C10^ii^	3.3094
S2···H13	3.0259	H3···C11^ii^	3.0861
S1···H9	2.721 (15)	H3···C12^ii^	3.0047
S1···H11	2.6961	H3···H11^ii^	2.6381
S1···H13	3.0558	H3···H13^ii^	2.6829
O1···H4	2.4918	H3···H15^ii^	2.2865
O1···H7	2.6484	H3···H16^ii^	3.3522
N1···H5	2.6194	H3···H26^iii^	3.4022
N2···H8	2.3679	H4···C4^xiv^	3.5050
C1···H7	2.5074	H4···C14^ii^	3.5310
C2···H1	2.5754	H4···C15^ii^	3.4923
C2···H2	2.6692	H4···H4^xiv^	3.5163
C2···H3	3.1945	H4···H5^xiv^	3.1607
C2···H5	3.2641	H4···H19^ii^	2.8569
C2···H6	3.2480	H4···H21^ii^	2.9400
C3···H7	3.2645	H4···H23^ii^	3.0754
C4···H6	3.2549	H4···H25^iii^	3.4102
C4···H8	3.3422	H4···H25^x^	2.9160
C5···H4	3.2702	H4···H26^iii^	3.5353
C5···H7	3.2784	H5···C16^x^	3.4872
C6···H5	3.2558	H5···C17^x^	3.0329
C6···H8	2.6377	H5···H2^iv^	3.1680
C7···H1	2.6779	H5···H4^xiv^	3.1607
C7···H2	2.8008	H5···H21^vi^	3.2685
C7···H4	3.2588	H5···H22^x^	2.9783
C8···H5	2.6856	H5···H25^x^	2.5007
C8···H6	2.6043	H5···H26^x^	2.8847
C8···H9	2.378 (17)	H6···C1^xii^	3.4304
C9···H11	2.7711	H6···C7^xii^	3.4668
C9···H13	3.2268	H6···C14^v^	3.2785
C10···H14	2.7366	H6···C16^v^	3.5292
C10···H15	2.6996	H6···H1^xii^	2.5789
C11···H16	2.6966	H6···H2^xii^	3.4703
C11···H17	2.7354	H6···H7^xii^	2.6947
C12···H10	2.6384	H6···H18^v^	3.2113
C12···H11	2.7870	H6···H19^v^	2.5304
C12···H18	2.7073	H6···H22^v^	3.3118
C12···H19	2.7613	H6···H23^v^	2.9820
C13···H12	2.7405	H6···H24^vi^	3.5408
C13···H13	2.7037	H7···C6^xii^	3.2430
C13···H20	2.7310	H7···C7^xii^	3.3134
C13···H21	2.7136	H7···H6^xii^	2.6947
C14···H14	2.7571	H7···H7^xii^	2.8520
C14···H15	2.7190	H7···H22^xiii^	3.1062
C14···H22	2.6894	H7···H23^xiii^	3.2518
C14···H23	2.8104	H7···H23^v^	3.4467
C15···H16	2.7538	H7···H24^iii^	3.5593
C15···H17	2.6999	H8···S1^i^	2.9639
C15···H24	2.6788	H8···C12^v^	3.4787
C15···H25	3.3623	H8···C14^v^	3.4473
C15···H26	2.7709	H8···H1^xii^	3.1650
C16···H18	2.7528	H8···H14^v^	3.2245
C16···H19	2.7357	H8···H15^v^	2.9005
C17···H20	2.6466	H8···H18^v^	3.0839
C17···H21	2.7707	H8···H19^v^	2.9561
H1···H7	2.2546	H8···H20^vi^	3.4994
H2···H7	2.3376	H8···H21^vi^	3.3796
H3···H7	3.4782	H9···S1^i^	2.535 (19)
H4···H5	2.3211	H9···N2^i^	3.437 (19)
H6···H7	2.3355	H9···C9^i^	3.47 (2)
H6···H8	2.4334	H9···C12^v^	3.537 (17)
H8···H9	2.1528	H9···H9^i^	2.84 (3)
H10···H12	2.4347	H9···H10^v^	3.3375
H10···H13	2.8577	H9···H14^v^	3.0126
H10···H14	2.4748	H9···H15^v^	3.1308
H10···H15	2.8433	H9···H16^vi^	3.5803
H11···H12	2.8584	H9···H17^vi^	3.1377
H11···H13	2.3179	H10···S1^vi^	2.9452
H11···H14	3.1276	H10···N2^vi^	3.3208
H11···H15	2.5935	H10···C9^vi^	3.0670
H12···H14	2.3686	H10···C13^x^	3.5527
H12···H15	2.8703	H10···C15^x^	3.5289
H12···H16	2.9920	H10···H9^vi^	3.3375
H12···H17	2.5710	H10···H13^vi^	3.1923
H13···H14	2.8703	H10···H17^x^	2.7303
H13···H15	2.3976	H10···H18^x^	3.3717
H13···H16	2.4898	H10···H20^x^	2.6850
H13···H17	2.9949	H11···S2^vi^	3.4182
H14···H16	2.8655	H11···N2^vi^	3.4566
H14···H17	2.3627	H11···C1^vii^	3.5543
H14···H18	2.5479	H11···C9^vi^	3.1237
H14···H19	3.0838	H11···C11^vi^	3.3213
H15···H16	2.3932	H11···H3^vii^	2.6381
H15···H17	2.8657	H11···H12^vi^	3.0549
H15···H18	2.9420	H11···H13^vi^	2.7593
H15···H19	2.5646	H12···S1^v^	3.1369
H16···H18	2.8632	H12···C9^v^	3.4048
H16···H19	2.3543	H12···C12^x^	3.3737
H16···H20	3.0518	H12···C13^x^	3.3534
H16···H21	2.5562	H12···C14^x^	3.3760
H17···H18	2.3977	H12···H11^v^	3.0549
H17···H19	2.8632	H12···H14^x^	2.6147
H17···H20	2.5169	H12···H17^x^	2.9670
H17···H21	2.9414	H12···H18^x^	2.6443
H18···H20	2.3650	H13···S2^v^	3.1108
H18···H21	2.8604	H13···C1^vii^	3.5770
H18···H22	2.5195	H13···C9^v^	3.4204
H18···H23	3.1319	H13···C10^v^	3.1786
H19···H20	2.8604	H13···H3^vii^	2.6829
H19···H21	2.3792	H13···H10^v^	3.1923
H19···H22	2.9180	H13···H11^v^	2.7593
H19···H23	2.6311	H14···S1^xvi^	3.4469
H20···H22	2.4246	H14···N2^vi^	3.3440
H20···H23	2.8587	H14···C11^x^	3.3834
H20···H24	2.8280	H14···C12^x^	3.3701
H20···H25	3.5700	H14···C13^x^	3.3332
H20···H26	2.5311	H14···H8^vi^	3.2245
H21···H22	2.8594	H14···H9^vi^	3.0126
H21···H23	2.3264	H14···H12^x^	2.6147
H21···H24	2.5621	H14···H14^x^	3.0159
H21···H26	3.1570	H14···H17^x^	2.5454
H22···H24	2.8570	H15···O1^vii^	2.8977
H22···H25	2.3908	H15···N1^vi^	3.1115
H22···H26	2.3335	H15···N2^vi^	3.0750
H23···H24	2.4045	H15···C1^vii^	2.8884
H23···H25	2.3211	H15···C8^vi^	3.0523
H23···H26	2.8557	H15···H2^vii^	3.1378
S2···H11^v^	3.4182	H15···H3^vii^	2.2865
S2···H13^vi^	3.1108	H15···H8^vi^	2.9005
S2···H16^vi^	3.4450	H15···H9^vi^	3.1308
S2···H18^x^	3.3864	H16···S2^v^	3.4450
S2···H20^x^	3.1271	H16···O1^vii^	3.1046
S2···H22^x^	3.2475	H16···N1^v^	3.0993
S2···H26^x^	3.5318	H16···N2^v^	3.2031
S1···H3^vii^	3.1229	H16···C9^v^	3.3082
S1···H8^i^	2.9639	H16···H3^vii^	3.3522
S1···H9^i^	2.535 (19)	H16···H9^v^	3.5803
S1···H10^v^	2.9452	H17···N1^v^	3.5600
S1···H12^vi^	3.1369	H17···N2^v^	3.1252
S1···H14^xi^	3.4469	H17···C9^v^	3.2922
O1···H15^ii^	2.8977	H17···C10^x^	3.4140
O1···H16^ii^	3.1046	H17···C11^x^	3.3522
O1···H19^ii^	2.9464	H17···C12^x^	3.3197
O1···H24^iii^	3.5092	H17···H9^v^	3.1377
O1···H26^iii^	2.8166	H17···H10^x^	2.7303
N1···H2^iv^	3.0576	H17···H12^x^	2.9670
N1···H15^v^	3.1115	H17···H14^x^	2.5454
N1···H16^vi^	3.0993	H18···S2^x^	3.3864
N1···H17^vi^	3.5600	H18···C10^x^	3.5590
N1···H21^vi^	3.2400	H18···C11^x^	3.4591
N2···H9^i^	3.437 (19)	H18···H2^xv^	3.5729
N2···H10^v^	3.3208	H18···H6^vi^	3.2113
N2···H11^v^	3.4566	H18···H8^vi^	3.0839
N2···H14^v^	3.3440	H18···H10^x^	3.3717
N2···H15^v^	3.0750	H18···H12^x^	2.6443
N2···H16^vi^	3.2031	H19···O1^vii^	2.9464
N2···H17^vi^	3.1252	H19···C3^vii^	3.4814
C1···H6^xii^	3.4304	H19···C5^vi^	3.4947
C1···H11^ii^	3.5543	H19···C6^vi^	3.0816
C1···H13^ii^	3.5770	H19···C8^vi^	3.3283
C1···H15^ii^	2.8884	H19···H4^vii^	2.8569
C1···H22^xiii^	3.3520	H19···H6^vi^	2.5304
C1···H26^iii^	3.2103	H19···H8^vi^	2.9561
C2···H24^iii^	3.3234	H20···S2^x^	3.1271
C2···H25^iii^	3.3303	H20···C8^v^	3.5758
C2···H26^iii^	3.1138	H20···C10^x^	3.3068
C3···H19^ii^	3.4814	H20···H8^v^	3.4994
C3···H25^iii^	3.2537	H20···H10^x^	2.6850
C3···H25^x^	3.4321	H21···N1^v^	3.2400
C3···H26^iii^	3.4990	H21···C4^v^	3.3174
C4···H2^iv^	3.3491	H21···C5^v^	3.1656
C4···H4^xiv^	3.5050	H21···C8^v^	3.0459
C4···H21^vi^	3.3174	H21···H4^vii^	2.9400
C4···H25^x^	3.2313	H21···H5^v^	3.2685
C5···H2^iv^	3.4202	H21···H8^v^	3.3796
C5···H19^v^	3.4947	H21···H25^ix^	3.5358
C5···H21^vi^	3.1656	H22···S2^x^	3.2475
C5···H24^vi^	3.5947	H22···C1^xv^	3.3520
C6···H1^xii^	3.2687	H22···H1^xv^	3.3830
C6···H7^xii^	3.2430	H22···H2^xv^	2.5239
C6···H19^v^	3.0816	H22···H5^x^	2.9783
C6···H24^vi^	3.4177	H22···H6^vi^	3.3118
C7···H6^xii^	3.4668	H22···H7^xv^	3.1062
C7···H7^xii^	3.3134	H23···C16^ix^	3.4170
C7···H24^iii^	3.4181	H23···C17^ix^	3.3270
C8···H2^iv^	3.3768	H23···H4^vii^	3.0754
C8···H15^v^	3.0523	H23···H6^vi^	2.9820
C8···H19^v^	3.3283	H23···H7^xv^	3.2518
C8···H20^vi^	3.5758	H23···H7^vi^	3.4467
C8···H21^vi^	3.0459	H23···H23^ix^	2.7337
C9···H9^i^	3.47 (2)	H23···H24^ix^	3.4671
C9···H10^v^	3.0670	H23···H25^ix^	2.7103
C9···H11^v^	3.1237	H24···O1^viii^	3.5092
C9···H12^vi^	3.4048	H24···C2^viii^	3.3234
C9···H13^vi^	3.4204	H24···C5^v^	3.5947
C9···H16^vi^	3.3082	H24···C6^v^	3.4177
C9···H17^vi^	3.2922	H24···C7^viii^	3.4181
C10···H3^vii^	3.3094	H24···C17^ix^	3.1111
C10···H13^vi^	3.1786	H24···H1^viii^	2.9558
C10···H17^x^	3.4140	H24···H6^v^	3.5408
C10···H18^x^	3.5590	H24···H7^viii^	3.5593
C10···H20^x^	3.3068	H24···H23^ix^	3.4671
C11···H3^vii^	3.0861	H24···H24^ix^	2.5751
C11···H11^v^	3.3213	H24···H25^ix^	2.8301
C11···H14^x^	3.3834	H25···C2^viii^	3.3303
C11···H17^x^	3.3522	H25···C3^viii^	3.2537
C11···H18^x^	3.4591	H25···C3^x^	3.4321
C12···H3^vii^	3.0047	H25···C4^x^	3.2313
C12···H8^vi^	3.4787	H25···C16^ix^	3.3726
C12···H9^vi^	3.537 (17)	H25···C17^ix^	3.3030
C12···H12^x^	3.3737	H25···H4^viii^	3.4102
C12···H14^x^	3.3701	H25···H4^x^	2.9160
C12···H17^x^	3.3197	H25···H5^x^	2.5007
C13···H10^x^	3.5527	H25···H21^ix^	3.5358
C13···H12^x^	3.3534	H25···H23^ix^	2.7103
C13···H14^x^	3.3332	H25···H24^ix^	2.8301
C14···H4^vii^	3.5310	H25···H25^ix^	3.2766
C14···H6^vi^	3.2785	H26···S2^x^	3.5318
C14···H8^vi^	3.4473	H26···O1^viii^	2.8166
C14···H12^x^	3.3760	H26···C1^viii^	3.2103
C15···H4^vii^	3.4923	H26···C2^viii^	3.1138
C15···H10^x^	3.5289	H26···C3^viii^	3.4990
C16···H2^xv^	3.4817	H26···H1^viii^	2.8571
C16···H5^x^	3.4872	H26···H3^viii^	3.4022
C16···H6^vi^	3.5292	H26···H4^viii^	3.5353
C16···H23^ix^	3.4170	H26···H5^x^	2.8847
C16···H25^ix^	3.3726		
			
C9—S2—C10	103.31 (7)	C5—C8—H8	119.226
C1—O1—C2	117.34 (13)	S2—C10—H10	108.816
N2—N1—C8	115.06 (13)	S2—C10—H11	108.818
N1—N2—C9	120.45 (12)	C11—C10—H10	108.829
O1—C2—C3	115.73 (14)	C11—C10—H11	108.819
O1—C2—C7	124.43 (13)	H10—C10—H11	107.692
C3—C2—C7	119.84 (13)	C10—C11—H12	109.164
C2—C3—C4	120.49 (15)	C10—C11—H13	109.167
C3—C4—C5	120.34 (13)	C12—C11—H12	109.165
C4—C5—C6	118.36 (13)	C12—C11—H13	109.183
C4—C5—C8	122.16 (12)	H12—C11—H13	107.873
C6—C5—C8	119.48 (14)	C11—C12—H14	109.164
C5—C6—C7	121.60 (15)	C11—C12—H15	109.150
C2—C7—C6	119.36 (13)	C13—C12—H14	109.145
N1—C8—C5	121.56 (14)	C13—C12—H15	109.144
S2—C9—S1	125.81 (8)	H14—C12—H15	107.864
S2—C9—N2	113.54 (11)	C12—C13—H16	108.911
S1—C9—N2	120.65 (12)	C12—C13—H17	108.908
S2—C10—C11	113.70 (10)	C14—C13—H16	108.897
C10—C11—C12	112.19 (12)	C14—C13—H17	108.910
C11—C12—C13	112.27 (12)	H16—C13—H17	107.734
C12—C13—C14	113.33 (12)	C13—C14—H18	108.957
C13—C14—C15	113.09 (12)	C13—C14—H19	108.962
C14—C15—C16	114.09 (13)	C15—C14—H18	108.966
C15—C16—C17	112.36 (14)	C15—C14—H19	108.970
N1—N2—H9	118.4 (11)	H18—C14—H19	107.759
C9—N2—H9	120.9 (11)	C14—C15—H20	108.728
O1—C1—H1	109.474	C14—C15—H21	108.736
O1—C1—H2	109.472	C16—C15—H20	108.725
O1—C1—H3	109.468	C16—C15—H21	108.741
H1—C1—H2	109.473	H20—C15—H21	107.632
H1—C1—H3	109.468	C15—C16—H22	109.138
H2—C1—H3	109.472	C15—C16—H23	109.119
C2—C3—H4	119.755	C17—C16—H22	109.139
C4—C3—H4	119.750	C17—C16—H23	109.125
C3—C4—H5	119.819	H22—C16—H23	107.857
C5—C4—H5	119.836	C16—C17—H24	109.476
C5—C6—H6	119.199	C16—C17—H25	109.469
C7—C6—H6	119.198	C16—C17—H26	109.467
C2—C7—H7	120.319	H24—C17—H25	109.485
C6—C7—H7	120.323	H24—C17—H26	109.469
N1—C8—H8	119.213	H25—C17—H26	109.462
			
C9—S2—C10—C11	83.88 (10)	C3—C4—C5—C6	0.0 (2)
C10—S2—C9—S1	3.71 (11)	C3—C4—C5—C8	−179.23 (12)
C10—S2—C9—N2	−176.36 (9)	C4—C5—C6—C7	0.3 (2)
C1—O1—C2—C3	−174.08 (13)	C4—C5—C8—N1	−10.9 (2)
C1—O1—C2—C7	5.0 (2)	C6—C5—C8—N1	169.89 (13)
N2—N1—C8—C5	176.89 (11)	C8—C5—C6—C7	179.53 (12)
C8—N1—N2—C9	173.58 (11)	C5—C6—C7—C2	−0.5 (3)
N1—N2—C9—S2	−4.84 (16)	S2—C10—C11—C12	169.73 (9)
N1—N2—C9—S1	175.10 (10)	C10—C11—C12—C13	177.45 (11)
O1—C2—C3—C4	178.82 (12)	C11—C12—C13—C14	−177.31 (12)
O1—C2—C7—C6	−178.47 (12)	C12—C13—C14—C15	−176.22 (12)
C3—C2—C7—C6	0.5 (2)	C13—C14—C15—C16	−178.79 (12)
C7—C2—C3—C4	−0.3 (3)	C14—C15—C16—C17	−171.39 (12)
C2—C3—C4—C5	−0.0 (3)		

Symmetry codes: (i) −*x*+3/2, −*y*+1/2, −*z*+1; (ii) *x*+1/2, −*y*+1/2, *z*+1/2; (iii) *x*+1/2, *y*−1/2, *z*+1; (iv) −*x*+2, −*y*+1, −*z*+1; (v) −*x*+3/2, *y*+1/2, −*z*+1/2; (vi) −*x*+3/2, *y*−1/2, −*z*+1/2; (vii) *x*−1/2, −*y*+1/2, *z*−1/2; (viii) *x*−1/2, *y*+1/2, *z*−1; (ix) −*x*+1, *y*, −*z*−1/2; (x) −*x*+3/2, −*y*+1/2, −*z*; (xi) *x*, −*y*, *z*+1/2; (xii) −*x*+2, *y*, −*z*+3/2; (xiii) *x*+1/2, *y*+1/2, *z*+1; (xiv) −*x*+2, −*y*, −*z*+1; (xv) *x*−1/2, *y*−1/2, *z*−1; (xvi) *x*, −*y*, *z*−1/2.

### Hydrogen-bond geometry (Å, º)

**Table:**

*D*—H···*A*	*D*—H	H···*A*	*D*···*A*	*D*—H···*A*
N2—H9···S1^i^	0.86 (2)	2.536 (18)	3.3870 (11)	170.9 (16)

Symmetry code: (i) −*x*+3/2, −*y*+1/2, −*z*+1.
