# Supplementary figures and images for: Crystal structure of S-octyl (E)-3-(4-meth­oxy­benzyl­idene)di­thio­carbazate
Source: Acta Crystallogr E Crystallogr Commun. 2015 Mar 28;71(Pt 4):o265–6. doi: 10.1107/S205698901500568X (PMC4438792; doi:10.1107/S205698901500568X)

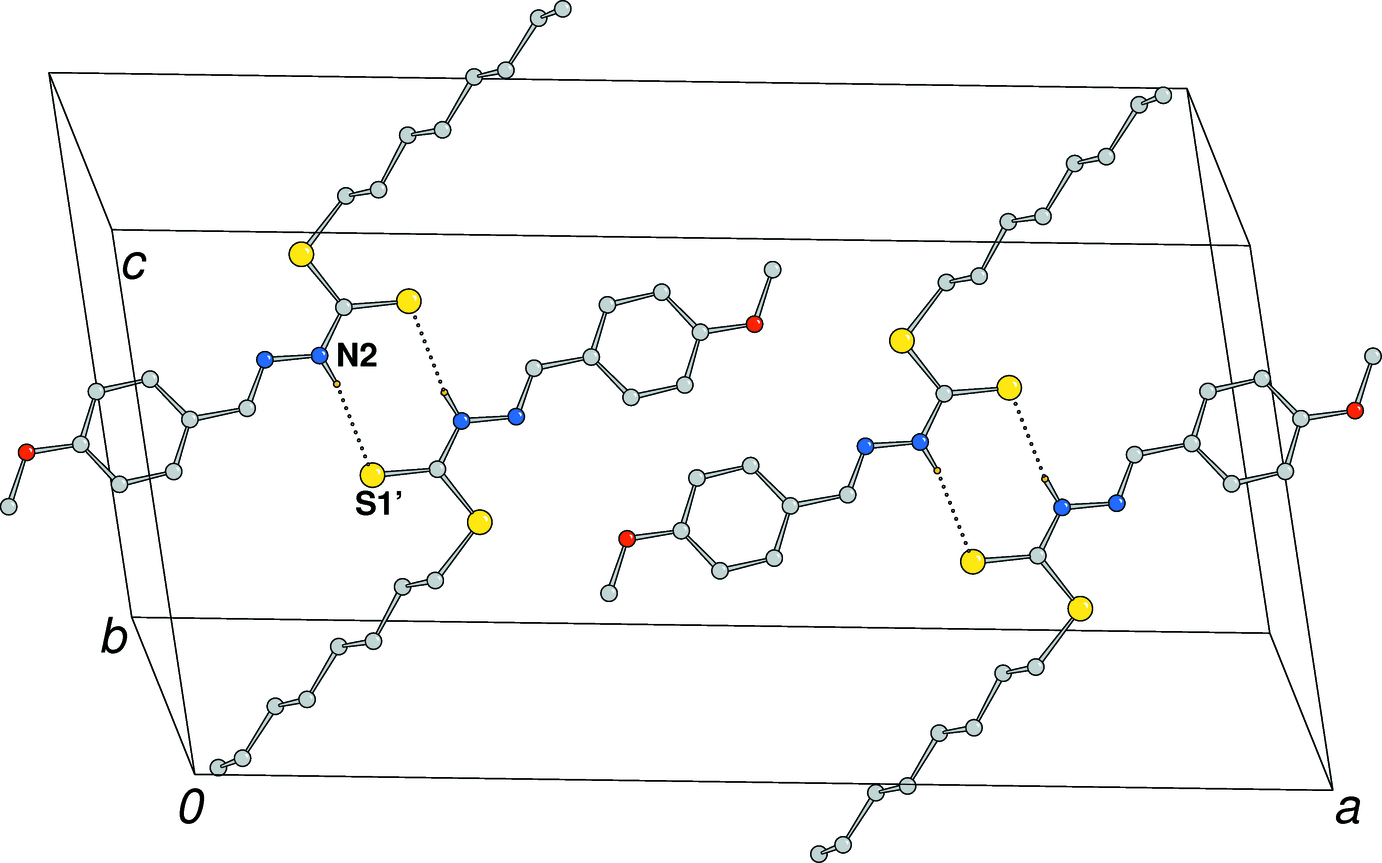

Supplement: Supplementary file 5 [file e-71-0o265-fig2.tif]
